# Identification of Two Flip-Over Genes in Grass Family as Potential Signature of C4 Photosynthesis Evolution

**DOI:** 10.3390/ijms241814165

**Published:** 2023-09-15

**Authors:** Chao Wu, Dianjing Guo

**Affiliations:** State Key Laboratory of Agrobiotechnology, School of Life Sciences, The Chinese University of Hong Kong, Shatin, New Territories, Hong Kong SAR, China; wuchao@link.cuhk.edu.hk

**Keywords:** C4 photosynthesis, comparative transcriptomics, transcriptome signature, Differentially Expressed Genes (DEGs), flip-over genes (FOGs), LHCA6, PGRL1A, AlphaFold2, sequence similarity, structure similarity, C4 traits engineering

## Abstract

In flowering plants, C4 photosynthesis is superior to C3 type in carbon fixation efficiency and adaptation to extreme environmental conditions, but the mechanisms behind the assembly of C4 machinery remain elusive. This study attempts to dissect the evolutionary divergence from C3 to C4 photosynthesis in five photosynthetic model plants from the grass family, using a combined comparative transcriptomics and deep learning technology. By examining and comparing gene expression levels in bundle sheath and mesophyll cells of five model plants, we identified 16 differentially expressed signature genes showing cell-specific expression patterns in C3 and C4 plants. Among them, two showed distinctively opposite cell-specific expression patterns in C3 vs. C4 plants (named as FOGs). The in silico physicochemical analysis of the two FOGs illustrated that C3 homologous proteins of LHCA6 had low and stable pI values of ~6, while the pI values of LHCA6 homologs increased drastically in C4 plants *Setaria viridis* (7), *Zea mays* (8), and *Sorghum bicolor* (over 9), suggesting this protein may have different functions in C3 and C4 plants. Interestingly, based on pairwise protein sequence/structure similarities between each homologous FOG protein, one FOG PGRL1A showed local inconsistency between sequence similarity and structure similarity. To find more examples of the evolutionary characteristics of FOG proteins, we investigated the protein sequence/structure similarities of other FOGs (transcription factors) and found that FOG proteins have diversified incompatibility between sequence and structure similarities during grass family evolution. This raised an interesting question as to whether the sequence similarity is related to structure similarity during C4 photosynthesis evolution.

## 1. Introduction

**Figure 1:**
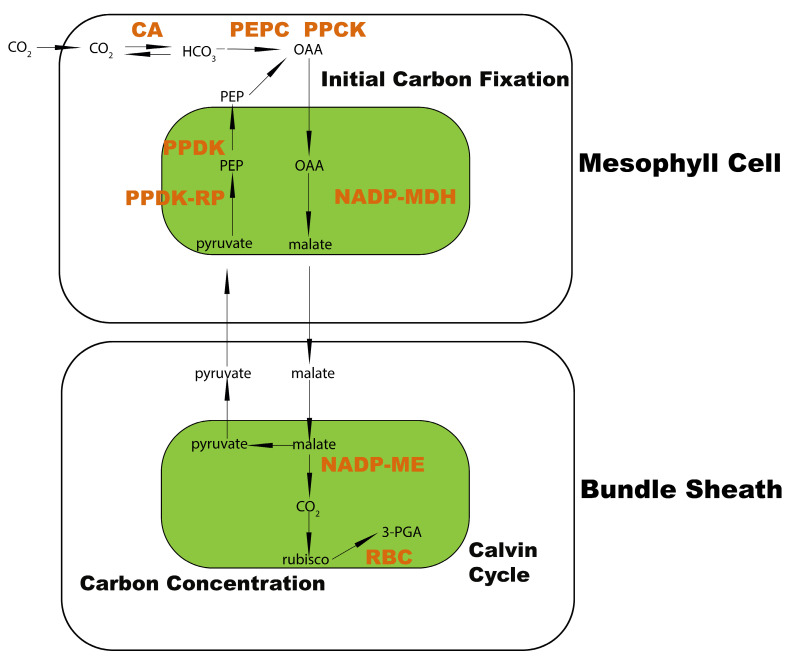
Typical NADP-ME subtype pathway illustration of C4 photosynthesis. Black frames represent two photosynthetic cells: bundle sheath and mesophyll. Green blocks are simplified chloroplasts. All carbon product names are in black, and all key C4 enzymes are in bold orange. Three vital processes of C4 photosynthesis are highlighted in bold black.

Photosynthesis is the ultimate energy source from solar power and supports most life forms on earth [1,2]. Based on the different initial carbon fixation processes, photosynthesis can be mainly classified into two subtypes: C3 photosynthesis and C4 photosynthesis [2]. Higher plants that carry out C4 photosynthesis have Kranz anatomy presented in the leaf tissue, hence the photorespiration process is prohibited. Plants therefore obtain more organic carbon and accumulate more biomass during the photosynthetic process, which allows them to adapt better to extreme environmental conditions such as heat and drought (Figure 1). This kind of adaptation has broad pleiotropic and epistatic consequences on C4 plants. For instance, they exhibit better water use efficiency and better heat tolerance [3,4]. In this case, certain unique genes have been connected to abiotic stress, implying that some key genes may serve as signatures for predicting the implications of sophisticated environmental stresses [5,6,7,8,9,10,11]. However, how C4 photosynthesis evolves from the ancestral C3 types remains unclear, especially whether the dynamics of gene expression is conserved among diverse higher plants. As we know, C4 photosynthesis has evolved independently in grass lineage [12]. The basis of plant evolution is the mutations of its DNA sequence. These mutations are reflected in the amino acid sequence divergence. Throughout the evolution of C4 photosynthesis, some proteins display a divergent expression, localization, and functionality [13], while others are more conserved. For example, the C4 enzyme coding genes are differentially presented between two photosynthetic cells, namely, mesophyll and bundle sheath. Some transcription factors and metabolite-related genes are also differentially regulated and undertake pivotal functions in the photosynthetic process [14,15]. All the differentially regulated genes compose the subtle differentiation of C4 photosynthesis.

As the first step to uncovering the mechanisms behind C4 photosynthesis evolution, studying the Differentially Expressed Genes (DEGs) between mesophyll and bundle sheath using transcriptome data has been a challenging task [16,17]. Dating back to the initial discovery of photosynthetic DEGs, scientists identified some key C4 enzymes differentially expressed in bundle sheath cells, such as PEP carboxylase and RuBisCO [18]. To decipher the gene transcripts accumulation in maize leaf blade, scientists separated the two photosynthetic cells and measured the differential gene expression using a microarray technique [19]. The maize leaf developmental gradient DEGs and cell-specific DEGs were also identified using Illumina sequencing [20]. To compare the transcriptome between C3 and C4 plants, scientists used developing leaves from maize and *Oryza sativa* (rice) and established a statistical model to simulate the changes between C3 and C4 plants during leaf development. *Setaria viridis* has recently been adopted as a new C4 model plant due to its simple genetics. For example, scientists combined comparative transcriptomics of diverse C4 plants including *Setaria viridis* and C3 rice to identify known and novel C4-related DEGs [12,21,22]. Some DEGs are photosynthetic genes that undertake key roles in various parts of photosynthesis [13,16,20,23]. To compare the C3 and C4 transcriptomes, the proper selection of the C3 model plant is pivotal. Scientists often choose panicoid grass for such study [22], whilst the consistency of cell-specific DEGs among different grass has not been discussed yet. Boosted by the subtle tissue separation technology such as laser microdissection, scientists successfully collected and sequenced the ultra-pure bundle sheath and mesophyll in two C3 plants, *Arabidopsis thaliana* and *Oryza sativa*, and identified new functions of bundle sheath from a physiology perspective [24,25]. The discoveries of known or novel DEGs in different sections such as developmental, cell-specific, or cross-species may help scientists narrow down the spectrum of C4 candidate genes for genetic engineering in C3 plants.

Among various types of DEGs, flip-over genes (FOGs) were defined as genes that display opposite cell-specific expression patterns between C3 and C4 plants. The first report about flip-over genes was from the comparison between maize (C4 NADP-ME) and *Cleome gynandra* (C4 NAD-ME), where 18 transcription factors were reported to show distinct expression preference between bundle sheath and mesophyll in maize and *Cleome gynandra* [26]. However, the authors did not reach further to investigate the structural basis of these FOGs.

Transcriptome signature discovery is a reliable approach to profiling gene expression during vital biological processes [27,28,29]. Generally, scientists focus on the conserved gene cascades and specifically expressed genes to meet the customized selective criteria [30]. In human biology studies, signature genes are well illustrated for further gene function studies [31]. In plant studies, however, few transcriptome signatures were identified due to limited data sets, especially for the photosynthesis study in the grass family [32].

As the end products of gene expression, proteins are often assembled as monomers or polymers to participate in diverse biological processes [33,34]. In recent years, structural scientists apply crystallography followed by X-ray or Nuclear Magnetic Resonance (NMR) [35,36] and Cryo-Electron Microscopy (Cryo-EM) to solve the structural folding of proteins of their interests [37]. Given that, the resources of universal protein folding information have been accumulated in the past decades [38]. The development of computational tools also promotes the discovery of protein three-dimensional structures [39], e.g., Swiss-model [40] and Phyre^2^ [41], etc. Unfortunately, the predictive capacity and accuracy of these tools are still limited [39,42].

In recent years, artificial intelligence (AI) has been widely applied in protein structure prediction. For example, AlphaFold2 platform achieved a median score of 92.4 GDT overall on the 14th Critical Assessment of Techniques for Protein Structure Prediction (CASP) assessment [43]. Its high performance was also demonstrated in protein complex prediction and peptide–protein docking in microorganism [44,45].

The sequence and structural similarities of proteins are often regarded as being equivalenced [46]. In terms of homologous proteins, the folds of proteins with sequence homology > 50% have close tertiary structures in general [47]. It is widely believed that the structure of proteins is more conserved than their sequence during evolution [48], or at least shows a linear relationship [49,50]. However, counterexamples that facilitate our limited knowledge about the protein sequence structure relationship diversity also exist. For example, some homologous CheY-like protein pairs with low sequence similarity (partial correlation coefficients were not statistically significant) exhibited very similar structural topology (based on distance matrix analysis of the C-terminal regions in native structures of these proteins) [51]. On the other hand, due to quaternary protein–protein interactions, some proteins with high pairwise sequence similarity (sequence identity ≥ 50%) presented largely diverged tertiary structural geometry and occupied 22% of the total sampled protein folds. This phenomenon has been discussed in TonB and ABL proteins from *E.coli* and *Drosophila melanogaster*, respectively [52], while it has not been reported in plants so far.

Nowadays, various bioinformatic tools have been developed to predict protein structure, function, and physicochemical properties [42,43,53,54]. These tools can facilitate our investigation of different proteins of our interests. Research about the molecular basis of the shifts from C3 to C4 photosynthesis has been well established in diverse corresponding phenotypes, such as leaf anatomy, chloroplast formation, and development of C4-specific Kranz anatomy, as well as the relevant regulatory genes that have been reported as clues to trace the trajectories of C4 evolution [14,55,56]. However, due to the uncoordinated homologous gene expression patterns or the low expression levels in our selected species, these genes cannot be utilized as reliable features to predict photosynthesis types. In addition, most machine learning-guided biomarker identifications start with a large gene expression matrix and end up with gene sets that pass the selective thresholds [57].

In this study, with the research hypothesis that cell-specific signature genes can be used to predict C4 photosynthesis type and that they have unique sequence/structure similarities from an evolutionary standpoint, we used the cutting-edge AlphaFold2 platform to predict the protein structures of homologous FOGs in five C3 and C4 model plants, and investigated the relationship between sequence and structure similarity. Our work expands our knowledge on C4 photosynthesis protein evolution, and possibly provide guidance for C4 photosynthesis engineering in C3 plants [58,59].

## 2. Results

### 2.1. Transcriptome Divergence between C3 and C4 Plants

**Figure 2:**
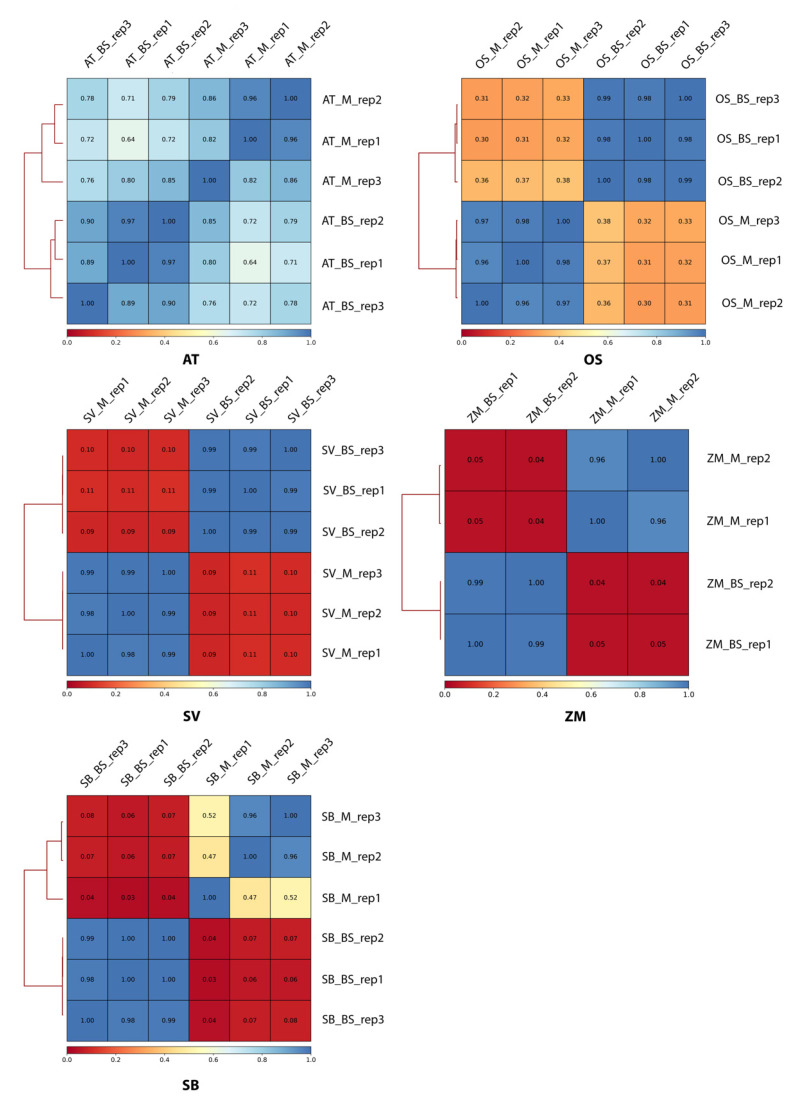
Internal consistency between each replicate of every selected photosynthetic model plant. Pearson correlation coefficient heatmap diagram of all collected replicates in five plants; a higher value represents higher consistency. All replicates are of high quality for reproduction except for the first replicate in SB, hence we removed it from the datasets in downstream analysis. We can see from the figure that the inter-cell-type consistency is well enough.

**Figure 3:**
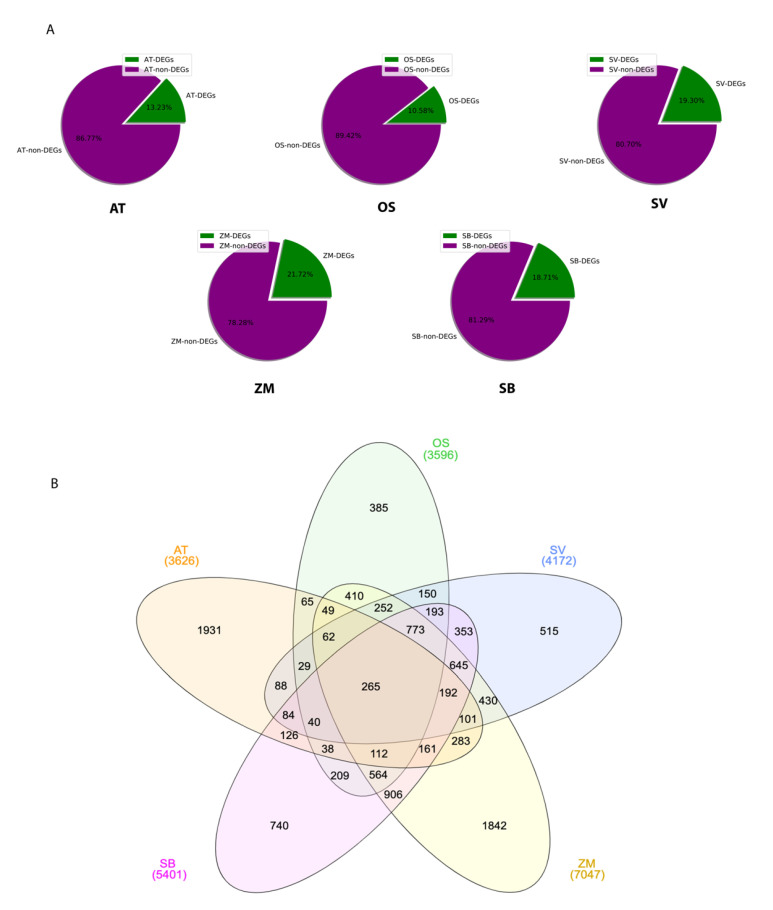
Comparative transcriptome analysis between C3 and C4 plants. (**A**) Cell-specific DEG proportions in each selected model plant. All data from five model plants are illustrated in pie charts. Purple represents non-DEGs, and green shows DEGs. (**B**) Multi-species Venn diagram of cell-specific DEGs from each model plant and their intersections in different sections. Among all five grass species, 265 C3 and C4 common DEGs are presented in the center of the Venn chart.

To comprehensively compare C3 and C4 transcriptomes, we investigated the high-quality replicates of cell-specific (bundle sheath and mesophyll) transcriptomes of five grass species, including *Arabidopsis thaliana* (C3), *Oryza sativa* (C3), *Setaria viridis* (C4), *Zea mays* (C4), and *Sorghum bicolor* (C4). Pearson correlation analysis showed high consistency between each replicate, indicating good reproducibility in general, except for the first replicate of *Sorghum bicolor*. We hence removed this replicate in further analysis (Figure 2). We analyzed the cell-specific differentially expressed genes (DEGs) and found that both C3 species presented a smaller portion of DEGs, compared to the three C4 species (Figure 3), suggesting the differentiation of bundle sheath and mesophyll has a greater impact on C4 transcriptomes as the formation of Kranz anatomy recruits vast differentially expressed genes in C4 plants. Among them, *Oryza sativa* and *Zea mays* presented the DEGs’ lowest and highest proportion of 10.58% and 21.72%, respectively (Figure 3A). We then analyzed the DEGs intersection among the five species and identified a group of 265 DEGs shared by both C3 and C4 species. Meanwhile, 645 DEGs shared by *Setaria viridis*, *Zea mays,* and *Sorghum bicolor* were identified as C4-specific ones (Figure 3B).

### 2.2. Common and Specific Pathways Enriched in C3 and C4 Plants

**Figure 4:**
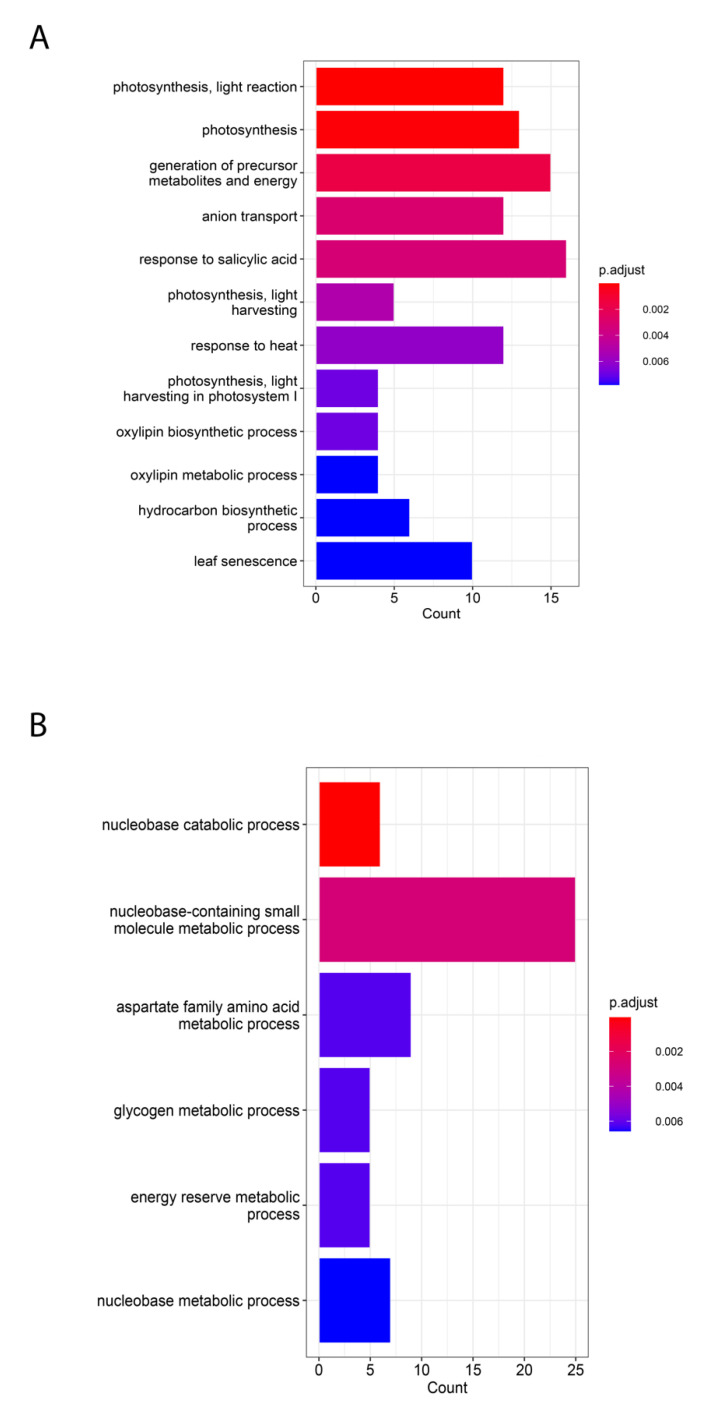
Gene ontology (GO) enrichment analysis of intersected DEGs. (**A**) GO enrichment analysis of C3 and C4 common DEGs. All twelve pathways are identified at the transcriptome-wide level from all five species. (**B**) GO enrichment analysis of C4-specific DEGs. All six pathways are identified at the transcriptome-wide level, and only from C4 species.

Based on our DEGs overlapping analysis results, we speculated that although evolutionary divergence exists between C3 and C4 plants, some genes remain active in both C3 and C4 species. The 265 common genes were mostly enriched in twelve pathways, e.g., the generation of precursor metabolites and energy, which is associated with the energy flow in photosynthesis; and hydrocarbon biosynthetic process, which is crucial for carbon transformation and delivery through photosynthesis. Apart from photosynthesis, C3 and C4 plants also have other divergent pathways in common, e.g., oxylipin biosynthetic and metabolic processes. In animals and humans, oxylipins act as pivotal precursors associated with diseases such as Alzheimer’s disease. In plants, oxylipins take part in the control of plant lifespan, reproductivity, and the defense to biotic stress [60] (Figure 4A). On the other hand, six C4-specific pathways (Figure 4B) were mainly associated with nucleobase metabolic and catabolic processes.

### 2.3. Transcriptome Signature Discovery in C3 and C4 Plants

**Figure 5:**
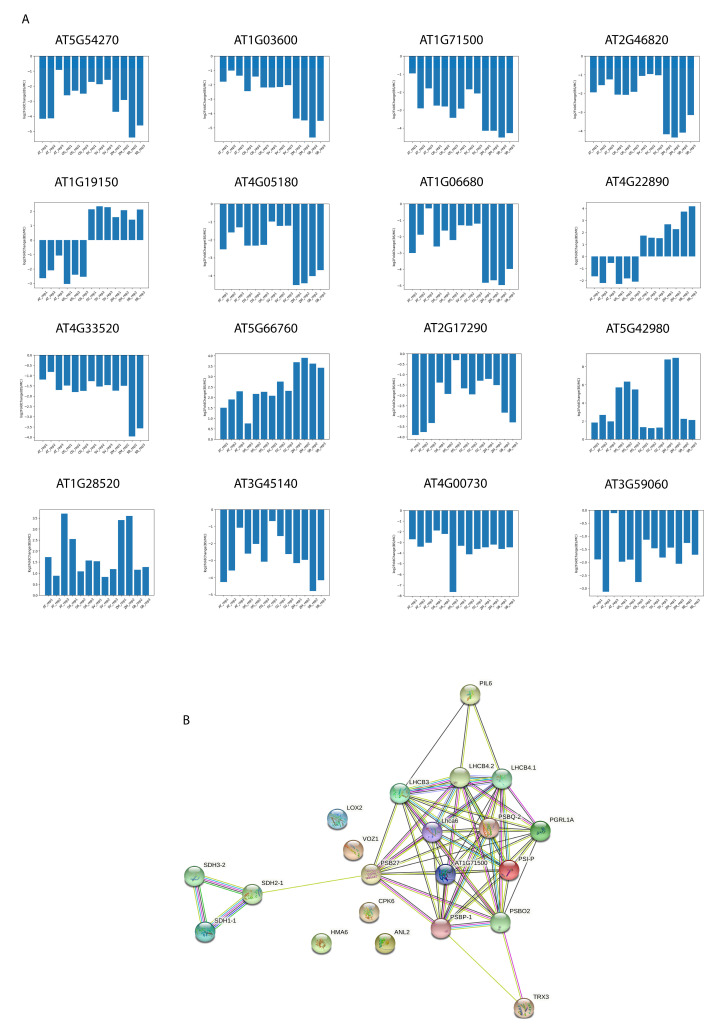
C3 and C4 transcriptome signature genes discovery. (**A**) Log2FoldChange values between bundle sheath and mesophyll of each replicate in five model plants. In each subplot, every column represents the log2FoldChange values. (**B**) Protein–protein interaction (PPI) prediction of 16 selected transcriptome signature genes. Each node shows a signature gene.

To gain knowledge about C3 and C4 plant convergence and divergence, 16 genes with cell-specific expression patterns were identified as C3 and C4 common signature genes. Figure 5A and Figure S1 showed the log2FoldChange values of these genes between bundle sheath and mesophyll of C3 and C4 plants. Among these signature genes, two flip-over genes (FOGs) showed opposite expression patterns in mesophyll and bundle sheath. When the protein–protein interactions among the signature genes were predicted (Figure 5B), we observed two subsections of interactive genes formed two weakly connected groups. One was the light-harvesting complex (LHC) for light energy capture [61], in which the two FOGs were included. Another was the tricarboxylic acid cycle (TCA cycle) for ATP synthesis [62].

### 2.4. Domain Analysis and Protein Folding Prediction of Two FOGs

**Figure 6:**
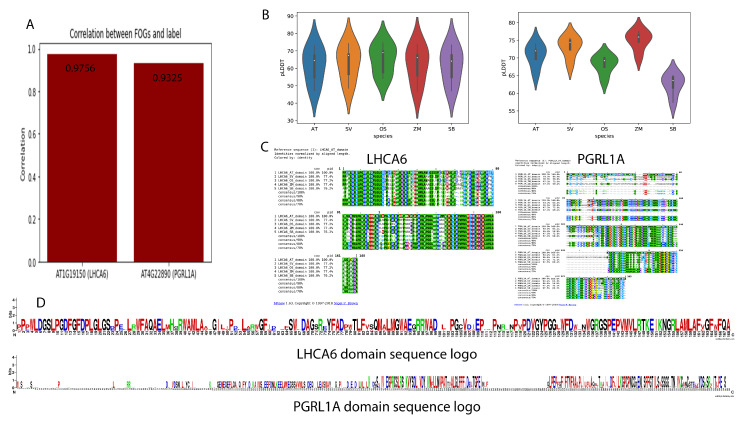
Domain analysis and protein three-dimension structure prediction metrics of two FOGs. (**A**) Pearson correlation between two FOGs and labels (species photosynthetic types). (**B**) pLDDT values of predicted structures from five species. (**C**) Domain sequence alignments of two FOGs in five model plants. (**D**) Domain sequence logos of two FOGs.

We examined the Pearson correlation coefficients against photosynthesis types (C3 or C4) and found that the cell-specific expression pattern of both FOGs were highly correlated with the photosynthetic types (over 0.9), indicating they may be used for photosynthetic type prediction (Figure 6A). We also retrieved the full sequences of FOG protein homologs in the five species and conducted protein folding prediction using AlphaFold2. To verify the performance of AlphaFold2 on plant protein structure prediction, we selected two plant proteins STP10 and ReAV, which were not included in the training dataset during the training process. The prediction accuracy was rather high as illustrated in Figure S2. Figure 6B illustrates the pLDDT values of each FOG protein structure prediction experiment. pLDDT is a metric for evaluating the prediction performance of AlphaFold2, and higher pLDDT means better prediction accuracy. AlphaFold2 achieved similar prediction performance on LHCA6 homologs. For membrane protein PGRL1A homologs, the pLDDT values were not stable. Since the sequence divergence of PGRL1A was much higher than that of LHCA6, it may also bring differences between each homolog. We then extracted the domain sequences of the two FOG protein homologs in five species and performed multiple sequence alignment (MSA) and visualized the amino acid mutations for all the selected protein domains. As shown, although the sequences were highly conserved, certain C4-specific mutations at amino acid level still existed (Figure 6C). From the sequence logo of the two FOG proteins (Figure 6D), the LHCA6 protein sequence was more conserved compared to PGRL1A. Interestingly, the consensus sequence for each protein was not continuous. We deduced that this is likely due to the genomic events that occurred during the course of evolution that break the original continuous sequence into segments. Such changes may facilitate the C4 plants’ adaptation to environmental cues.

### 2.5. Amino Acids Composition Analysis and Protein Solubility Prediction of FOGs

**Figure 7:**
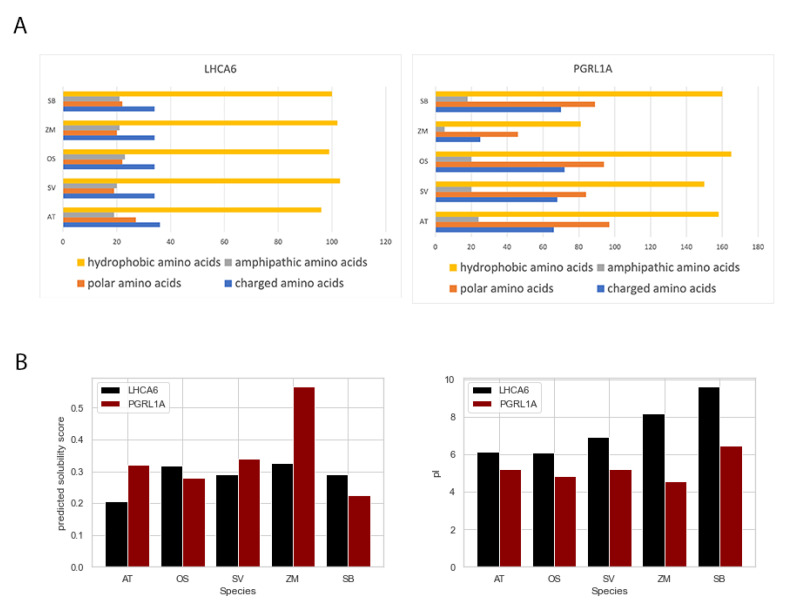
Amino acids composition analysis and protein solubility prediction of two FOGs. (**A**) Amino acids composition analysis based on protein domain sequences of two FOGs. (**B**) Protein solubility and pI value prediction of two FOGs.

The amino acid compositions of each FOG homolog domain were examined. The amino acids were characterized into four groups based on their physicochemical properties, including hydrophobic amino acids, amphipathic amino acids, polar amino acids, and charged amino acids. Most proteins examined consisted of hydrophobic amino acids that facilitate protein folding into a relatively stable conformation and maintain relevant functions (Figure 7A), followed by charged amino acids. In *Zea mays,* PGRL1A contained the fewest number of charged amino acids associated with the lowest pI value (Figure 7B). Polar amino acids are highly associated with protein solubility, and we found that they were correlated with protein solubility of LHCA6. This also held true for PGRL1A proteins, except for the *Zea mays* homolog. Furthermore, we found the pI values for LHCA6 protein in C3 plants AT and OS were relatively low and stable. While in C4 plants SV, ZM, and SB, the pI values increased drastically.

### 2.6. Sequence Motif Discovery in FOGs

**Figure 8:**
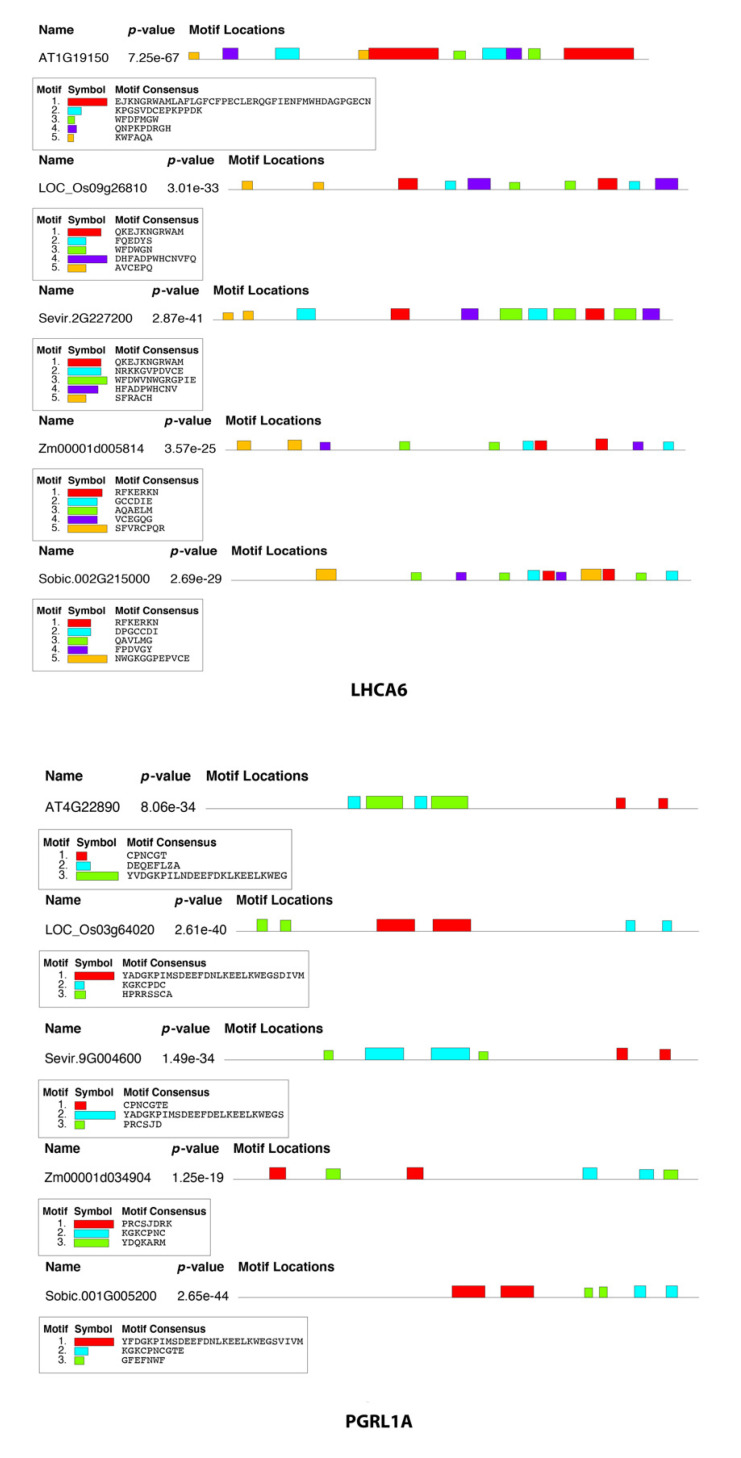
Sequence motif discovery of two FOGs. All five homologs of LHCA6 are listed on the left panel, and on the right panel are PGRL1A homologs. Consensus sequences are listed in the chart.

To investigate the changes in FOG proteins during C4 photosynthesis evolution, we conducted sequence motif analysis for each homologous protein. The top-ranked motif in *Arabidopsis thaliana* LHCA6 was annotated as a shorter consensus motif in *Oryza sativa* (Figure 8). The third consensus motif that begins with “WFD” also presented in *Oryza sativa* and *Setaria viridis* in different length and composition, but not in *Zea mays* and *Sorghum bicolor*. This indicated that *Setaria viridis* is likely more closely related to C3 plants compared to *Zea mays* and *Sorghum bicolor*. Furthermore, between *Zea mays* and *Sorghum bicolor*, the top-ranked motif “RFKERKN” appeared twice in both plants. Unlike LHCA6, the consensus motif identified in PGRL1A was less. The last-ranked motif consensus in *Arabidopsis thaliana* was also presented in *Oryza sativa*, *Setaria viridis,* and *Sorghum bicolor* with amino acid substitutions, but not in *Zea mays*. For maize, only short motif consensus was identified.

### 2.7. Sequence and Structure Similarities Comparison of FOG Proteins

**Figure 9:**
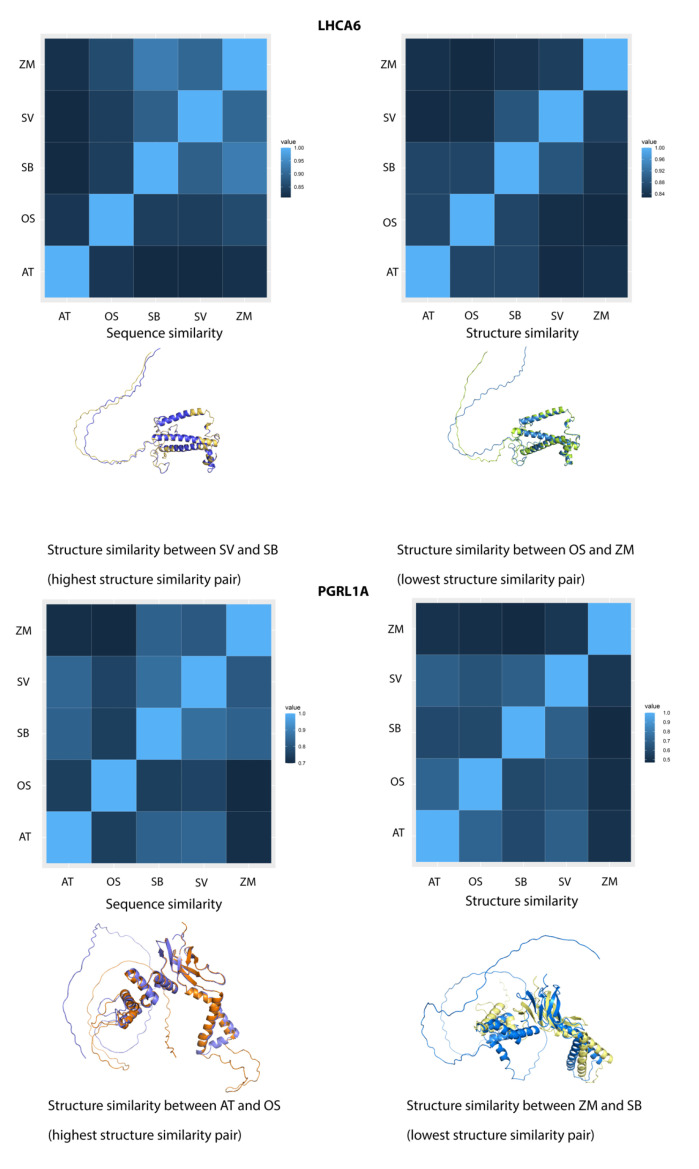
Sequence and structure similarities of two FOGs. The left panel is a similarity illustration of LHCA6, and another part is for PGRL1A. Each block in the heatmaps represents a pairwise comparison between homologous proteins. The brighter the color means the higher the similarity score.

For LHCA6, both global sequence and structure similarities were over 0.8. The consistent dual similarities showed consistency with the grass family phylogeny (Figure 9). We extracted the highest (SV-SB) and lowest (OS-ZM) structure-similarity pairs with similarity values of 0.88655 and 0.82752, respectively. Compared to the C3-C4 pair, the C4-C4 pair had closer phylogenetic relationship and hence a higher structure similarity. Moreover, we also found that the secondary structures in both pairs aligned very well, while the intrinsically disordered regions (IDRs) were differentially aligned. Precisely, the IDRs in the SV-SB pair were physically close. However, the spatial positions of the IDRs diverged in the OS-ZM pair. This finding indicated that when the global structure-similarity was high and secondary structures aligned very well, the IDRs would largely affect the overall structure similarity.

On the other hand, the sequence and structure similarities of PGRL1A were over 0.7 and 0.5, respectively. In Figure 8, the AT-OS pair shared a sequence similarity value of 0.75, while for AT-SB and AT-SV pairs the values were 0.83 and 0.84, respectively. Accordingly, the structure similarity of these three pairs were 0.71106, 0.60251, and 0.69203, respectively. As we extracted the structure alignments of the AT-OS (highest structure similarity) and the ZM-SB (lowest structure similarity) pairs, we found the alignment of secondary structures between AT and OS was very good. While in the ZM and SB pair, the structural alignment of secondary structures was drastically shifted and the TM-score was only 0.47017, suggesting the protein folds were not similar and the homology relationship was weak although they shared a high sequence similarity of 0.83 (Figure 9). From the case of PGRL1A, we found that the sequence and structure similarities showed local inconsistency.

It is widely assumed that structural features are often closely related to sequence composition. As reported by previous research, protein pairs with sequence identity higher than 35–40% are very likely to be globally structurally similar as well. Our findings provided refinements of this assumption that local inconsistency may affect the sequence and structure relationship. To further validate our finding, we selected three transcription factor type FOG proteins from a previous study by Aubry et al., namely SIGC, ZFP8, and ZHD10. Unlike ZFP8, both sequence and structure similarities of SIGC and ZHD10 were over 0.5, while the local comparison in SIGC was quite interesting. Precisely, we found that even if the sequence similarities between AT-SV/ZM (over 0.5) and OS-SV/ZM (over 0.7) were different, their structure-similarities were quite similar (over 0.5), similar to the situation in PGRL1A. In addition, although the ZFP8 protein pairs showed inconsistent sequence (over 0.5) and structure (over 0.2) similarities, the global homology relationship was maintained based on the two similarity heatmaps (Figure S3). We therefore conclude that FOG proteins have diversified incompatibility between sequence and structure similarities during grass family evolution, especially for the pairs that share low/diverged sequence similarity but have high/similar local structure similarity.

We then selected the top three transcription factor type non-FOG proteins from Aubry et al. for comparison, namely EFM, RL6, and SIGB. We also observed that EFM proteins had high sequence similarity (over 0.5) while showing drastically low global structure similarity (over 0.19), which suggested that non-FOG proteins also present highly diverged three-dimensional structures due to the complexity of protein evolution (Figure S4).

## 3. Discussion

It is known that C4 photosynthesis evolved independently [12], while the evolutionary events that occurred during the process have not been clarified. In this study, we compared the transcriptomes between C3 and C4 plants, and identified 16 signature genes differentially expressed between mesophyll and bundle sheath cells. Using differentially expressed genes as biomarkers to predict specific diseases is a commonly used bioinformatics strategy. Such analysis can narrow down the unique genes that are closely connected with targeted biological process or treatments. When we selected the DEGs, all candidate genes should show differential expression patterns in all selected species. Based on such criteria, only two FOGs are identified. Using the cutting-edge deep learning model AlphaFold2 and other protein informatics tools, we analyzed the sequences and structures of the two FOG proteins and found that local sequence and structure similarities showed inconsistency. This finding was consistent with previous reports [34,63,64]. However, from the comparison of sequence and structure similarities, we still identified novel structural divergence between homologous proteins, especially for the rearrangements of secondary structures in PGRL1A proteins.

Prediction of protein three-dimensional structure has been a crucial biological and computational challenge for the past few decades [43]. Deep learning technology utilizes numerous protein folding data to establish prediction models based on amino acid sequences. In recent years, AlphaFold2 has played a pivotal role in new structure discovery [43] with its well-established prediction platform based on Google Colaboratory [65]. We adopted Amber relaxation and templates during the prediction process to maximize the prediction performance. Through verification of the performance on plant protein prediction, our results indicated that AlphaFold2 was robust and reliable.

Signature genes shared by C3 and C4 plants have great potential to serve as the predictive features for classification tasks from a machine-learning perspective. In this study, we successfully identified 16 C3 and C4 signature genes in the grass family, including two FOGs. Compared to LHCA6, PGRL1A seems to be more active during plant evolution. PGRL1A is the hub gene of electron transport in photosynthesis [66,67]. The highly variant structures among homologous PGRL1A proteins may indicate the complex evolution of electron transport process from C3 to C4 plants. LHCA6 is the key component of the light-harvesting complex [68,69]. Divergence in its structure may directly affect the effectivity and efficiency of solar energy capture by altering the binding affinity and specificity, and results in the biomass accumulation differences between C3 and C4 plants. Our study suggested that residue preference may occur during the folding of FOG proteins, which may contribute to the diverse functionalities. In terms of homologous species, our results investigated their evolutionary relationship in three layers, namely, gene expression level, motif occurrence, and protein structure similarity. From the first two layers, *Setaria viridis* is closer to *Arabidopsis thaliana* and *Oryza sativa*, rather than *Zea mays* and *Sorghum bicolor*, while in protein structure similarity comparison of LHCA6, a highly conserved protein, *Setaria viridis* seems to be closer to *Sorghum bicolor*. This phenomenon raised the great importance of whether point mutations will drastically affect the protein structure and the corresponding measurements and provide a good initiation to investigate with large-scale samples in the future. As reported, point mutations may cause diverged functions of proteins translated from genes that have special expression patterns [70]. And our protein structure similarity navigation coordinates well with the previous findings in mice. Based on our findings, as FOGs are differentially expressed between bundle sheath and mesophyll in C3 and C4 species, an overview of their cross-species subcellular localization may provide an important clue as to whether the shifts in their expression patterns and three-dimensional structures contribute to their functional divergence [71]. Moreover, a precise location of the point mutations would help explain the structural divergence. For proteins like the bHLH transcription factors, the key mutations will affect the structure similarity drastically if they occur in the loop region. For the FOG proteins identified in our study, both are membrane proteins. Compared to the selected transcription factor type FOG proteins, they have higher global sequence similarity. For now, we are not sure whether it is due to certain protein types or the different evolutionary backgrounds.

It must be stated that our hypothesis was based on structural data generated by computational prediction and may not always reflect the natural protein folding. Secondly, plant evolution depends on multiple evolutionary events such as point mutations, chromosomal sequence alterations and number changes. Navigating plant evolution at the genomic level (such as single nucleotide polymorphism) and at the transcriptomic level (such as differential gene expression) may anchor different gene sets for making predictions. Our study thus only gave a glimpse of photosynthesis evolution in higher plants as a convolution method.

## 4. Perspective

How to better integrate comparative in silico gene evolutionary analysis to assess gene diversity across species remains a great challenge in multi-omics-based systems biology. In terms of its application in plant breeding and genetic engineering of specific metabolism processes such as photosynthesis, different layers of information may serve as guidance for genetic modification. The predictive genomic approaches can boost the detection of allelic-level variants of a single gene [72,73,74]. In this study, we mainly focus on the expression divergence of several genes related to C4 photosynthesis. We aligned the identified FOGs to their homologous proteins, whilst the genomic variants of these genes have not been discovered. To gain a comprehensive knowledge of these signature genes, integration of genomics, transcriptomics, and proteomics data from homologous species collected from diverse ecological regions may facilitate our understanding of protein evolution in C4 photosynthesis.

## 5. Materials and Methods

### 5.1. Comparative Transcriptome Analysis between C3 and C4 Plants

RNA-Sequencing data were retrieved from the European Nucleotide Archive under project accession PRJNA668247 (https://www.ebi.ac.uk/ena/browser/view/PRJNA668247?show=reads) (accessed on 12 September 2023) [24], PRJNA673407 (https://www.ebi.ac.uk/ena/browser/view/PRJNA673407?show=reads) (accessed on 12 September 2023) [25], and PRJEB5074 (https://www.ebi.ac.uk/ena/browser/view/PRJEB5074?show=reads) (accessed on 12 September 2023) [75] for C3 plant *Arabidopsis thaliana*, *Oryza sativa,* and C4 plant *Setaria viridis,* respectively. C4 plants *Zea mays* and *Sorghum bicolor* transcriptome data were retrieved from NCBI under accessions SRP009063 (https://www.ncbi.nlm.nih.gov/sra/?term=SRP009063) (accessed on 12 September 2023) [76] and PRJEB11652 (https://www.ncbi.nlm.nih.gov/sra/?term=PRJEB11652) (accessed on 12 September 2023) [77]. Raw reads were analyzed by FastQC (https://www.bioinformatics.babraham.ac.uk/projects/fastqc/) (accessed on 12 September 2023) for quality control and trimmed by Trimmomatic (http://www.usadellab.org/cms/?page=trimmomatic) (accessed on 12 September 2023) [78]. The reference genomes were downloaded via JGI Phytozome as *Arabidopsis thaliana* TAIR10 (https://data.jgi.doe.gov/refine-download/phytozome?genome_id=167) (accessed on 12 September 2023) [79], *Oryza sativa* v7.0 (https://data.jgi.doe.gov/refine-download/phytozome?genome_id=323) (accessed on 12 September 2023) [80], *Setaria viridis* v2.1 (https://data.jgi.doe.gov/refine-download/phytozome?genome_id=500) (accessed on 12 September 2023) [81], *Zea mays* B73 RefGen_v4 (https://data.jgi.doe.gov/refine-download/phytozome?genome_id=493) (accessed on 12 September 2023) (https://phytozome-next.jgi.doe.gov/info/Zmays_RefGen_V4) (accessed on 12 September 2023), and *Sorghum bicolor* v3.1.1. (https://data.jgi.doe.gov/refine-download/phytozome?genome_id=454) (accessed on 12 September 2023) [82]. HISAT2 (http://daehwankimlab.github.io/hisat2/) (accessed on 12 September 2023) was used for sequencing reads alignment to reference genomes [83]. The output files conversion from SAM to BAM format was performed by Samtools (https://www.htslib.org/) (accessed on 12 September 2023) [84]. The sorted and indexed BAM files were processed by the plotcorrelation function from deepTools (https://deeptools.readthedocs.io/en/develop/) (accessed on 12 September 2023) to analyze the internal consistency between replicates [85]. The following reads count step was processed by htseq-count from HTSeq 0.11.1 (https://htseq.readthedocs.io/en/release_0.11.1/count.html) (accessed on 12 September 2023) [86], and the count tables were passed to DESeq2 (https://bioconductor.org/packages/release/bioc/html/DESeq2.html) (accessed on 12 September 2023) for differentially expressed genes analysis [87]. Genes with adjusted *p*-value < 0.05 and the absolute value of log2FoldChange between bundle sheath and mesophyll > 1 were identified as the differentially expressed genes (DEGs) for further analysis. The overlapped DEGs between all five model plants were intersected and plotted by interactivenn (http://www.interactivenn.net/) (accessed on 12 September 2023) [88]. Among them, only C3 and C4 common DEGs and C4-specific DEGs were annotated by clusterProfiler (https://bioconductor.org/packages/release/bioc/html/clusterProfiler.html) (accessed on 12 September 2023) using gene ontology terms with *p*-value < 0.01 and q value < 0.05 [89]. To identify transcriptome signature genes, we calculated the log2FoldChange value for each pair of expressions in bundle sheath and mesophyll of DEGs in all twelve pathways enriched as C3 and C4 common. The DEGs with null expression were removed, and the DEGs showing similar expression patterns were kept as the signature genes to identify the features of photosynthesis. Among them, two DEGs that showed opposite cell-specific expression were characterized as flip-over genes (FOGs). In total, 20 transcriptome signature genes between the C3 and C4 species were identified. The visualization of DEGs proportion and the plot of log2FoldChange values between bundle sheath and mesophyll of transcriptome signature genes was performed by Microsoft Excel, python matplotlib (https://matplotlib.org/) (accessed on 12 September 2023) and R version 4.0.4 (https://www.r-project.org/) (accessed on 12 September 2023).

### 5.2. Protein-Protein Interaction Prediction of C3/C4 Transcriptome Signature Genes

Using STRING, we predicted the putative protein–protein interaction relationship between the selected 16 transcriptome signature proteins (https://string-db.org/) (accessed on 12 September 2023). Each node represents a signature gene, and the edge connecting two nodes represents the interactive relationship between two genes. More edges between two nodes indicate higher confidence.

### 5.3. Multiple Sequence Alignment for Specific Domains and FOGs Expression Pattern

To identify the domain sequences in each FOG protein, we used Interpro (https://www.ebi.ac.uk/interpro/) (accessed on 12 September 2023) and performed multiple sequence alignment (MSA) using MEGA X [90]. The MSA results were visualized using MView (https://www.ebi.ac.uk/Tools/msa/mview/) (accessed on 12 September 2023). We used weblogo (https://weblogo.berkeley.edu/logo.cgi) (accessed on 12 September 2023) to create the sequence logo for each protein domain [91]. Pearson correlation between log2FoldChange values of FOGs and the corresponding plant photosynthetic types (C3 or C4) was calculated using python pandas (https://pandas.pydata.org/) (accessed on 12 September 2023).

### 5.4. Domain Amino Acids Composition Analysis, Protein Solubility Prediction, and Global Motif Discovery

We identified and extracted each FOG protein’s domain sequences and analyzed their amino acid composition using ProtParam (https://web.expasy.org/protparam/) (accessed on 12 September 2023) from Expasy, aiming to compare the dynamics of amino acids with different characteristics during evolution. In addition, we utilized Protein-Sol (https://protein-sol.manchester.ac.uk/) (accessed on 12 September 2023) [54] to predict the solubility and pI of each protein. The motif occurrence in each homologous FOG protein was examined by MEME suite (https://meme-suite.org/meme/tools/meme) (accessed on 12 September 2023) [92].

### 5.5. Three-Dimensional Structure Prediction and Sequence Structure Similarity Comparison

To verify the prediction performance of AlphaFold2 in plant protein prediction, protein amino acid sequences and three-dimensional structures of plant protein STP10 and ReAV were retrieved from RCSB PDB with accessions 6H7D (https://www.rcsb.org/structure/6H7D) and 7OS5 (https://www.rcsb.org/structure/7OS5), (accessed on 12 September 2023) respectively. The structure predictions of the two proteins were generated by the ColabFold (https://colab.research.google.com/github/sokrypton/ColabFold/blob/main/AlphaFold2.ipynb) (accessed on 12 September 2023) with a substitution of MSA using MMseqs2 [65] and visualized by PyMol (https://pymol.org/2/) (accessed on 12 September 2023), and TM-score values were calculated by TM-align (https://zhanggroup.org/TM-align/) (accessed on 12 September 2023) [93]. Besides this, all monomers in five species of two FOG proteins were predicted using their amino acid sequences with Amber relaxation and templates on NVIDIA Tesla V100 GPU via the Google Colaboratory Pro+ platform. We collected five predicted protein folds for each input and plotted the pLDDT value using seaborn (http://seaborn.pydata.org/) (accessed on 12 September 2023). The models with the highest pLDDT were selected as the predicted model for structure comparison. The TM-score (https://zhanggroup.org/TM-score/) (accessed on 12 September 2023) and the Root Mean Square Deviation (RMSD) of superposition between predicted protein folds were calculated. TM-score is a classical measurement for pairwise protein structure topological similarity comparison, in which 1 represents the same fold, a value below 0.17 represents randomly selected unrelated structures, and 0.5 is the threshold for similar structure measurements. Generally, TM-score is regarded as a more sensitive measurement since it is length-dependent and it normalizes the distance errors, hence it can supplement RMSD [94]. We used the blastp program (https://blast.ncbi.nlm.nih.gov/Blast.cgi?PROGRAM=blastp&PAGE_TYPE=BlastSearch&LINK_LOC=blasthome) (accessed on 12 September 2023) to compare the sequence similarity of homologous proteins among five plant species. The dual similarities comparison method is well illustrated and utilized in protein studies [51,95,96].

## 6. Conclusions

In this work, we compared the cell-specific (bundle sheath and mesophyll cells) transcriptomes of five photosynthetic plant species. Our study illustrates that: (i) Compared to C3 plants, C4 plants have more cell-specific DEGs, which means that a more complex functional differentiation may occur in C4 plants. (ii) Among these cell-specific DEGs, we found 16 of them can be used as photosynthetic features for modeling. (iii) Two flip-over genes, LHCA6 and PGRL1A, are highly correlated with C3 or C4 photosynthetic types, which is due to their functional nature in the photosynthesis process. (iv) Based on protein physicochemical and structural analysis, we found the homologous proteins of these two flip-over genes are inconsistent in terms of their sequence and structure similarities, which are also found in other photosynthetic proteins, and may contribute to our understanding of the complexity of protein evolution in C4 photosynthesis.

## Data Availability

Not applicable.

## Supplementary Materials

The following supporting information can be downloaded at: https://www.mdpi.com/article/10.3390/ijms241814165/s1.
