# Supplementary material for: Identification of Two Flip-Over Genes in Grass Family as Potential Signature of C4 Photosynthesis Evolution
Source: Int J Mol Sci. 2023 Sep 15;24(18):14165. doi: 10.3390/ijms241814165 (PMC10531853; doi:10.3390/ijms241814165)
Supplement: Supplementary file 1 [file ijms-24-14165-s001.zip › ijms-2523235-supplementary.pdf]

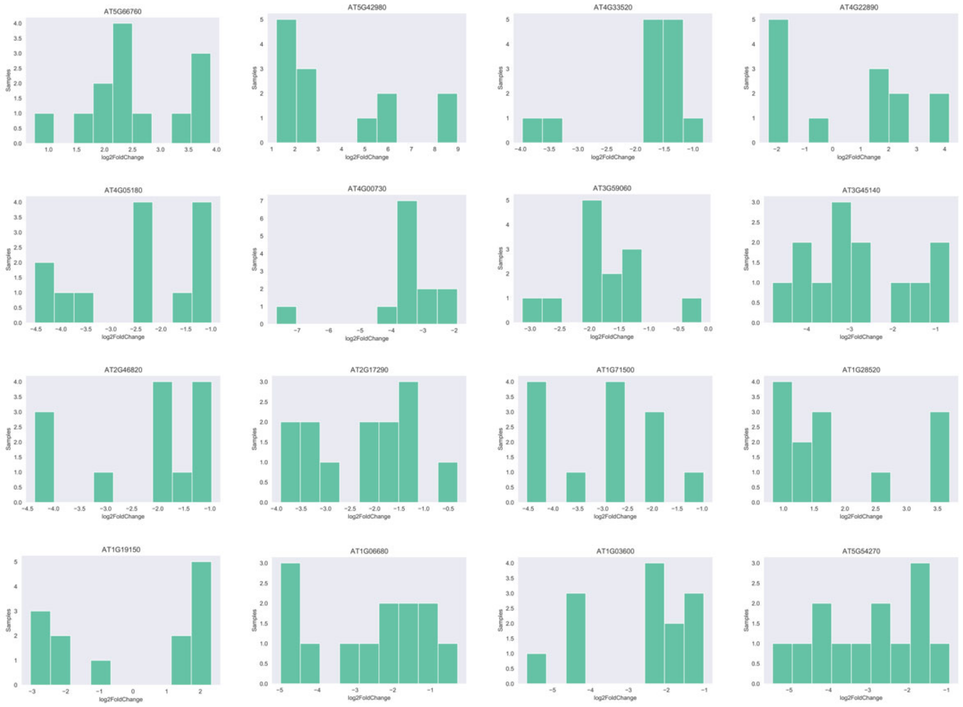

Figure S1. The detailed expression pattern of C3 and C4 transcriptome signature genes. Horizontal axes represent each expression pair between bundle sheath and mesophyll in five model plants. Vertical axes show the number of samples in each log2 FoldChange value.

A

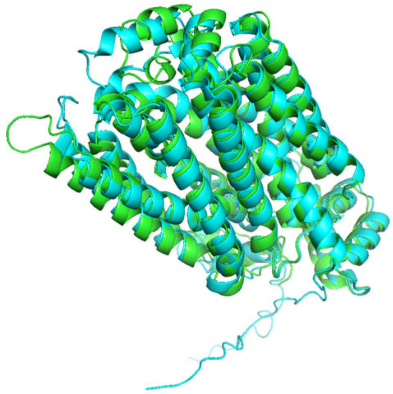

STP10 predicted fold and experimental fold comparison

Green is the experimental fold and blue is the predicted fold

TM-score = 0.94967 (if normalised by length of Chain_1)

TM-score = 0.88965 (if normalised by length of Chain_2)

RMSD = 1.304

B

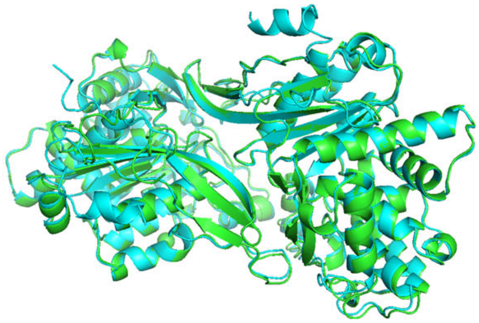

ReAV predicted fold and experimental fold comparison

Green is the experimental fold and blue is the predicted fold

TM-score = 0.95137 (if normalised by length of Chain_1)

TM-score = 0.93112 (if normalised by length of Chain_2)

RMSD = 0.447

Figure S2. AlphaFold2 prediction performance verification with plant protein folds. (**A**) Comparison between predicted and experimental structures of STP10. TM-scores between them are 0.94967 and 0.88965 normalized by chain 1 and chain 2, respectively. Moreover, the RMSD value is 1.304. (**B**) Comparison between the predicted and.

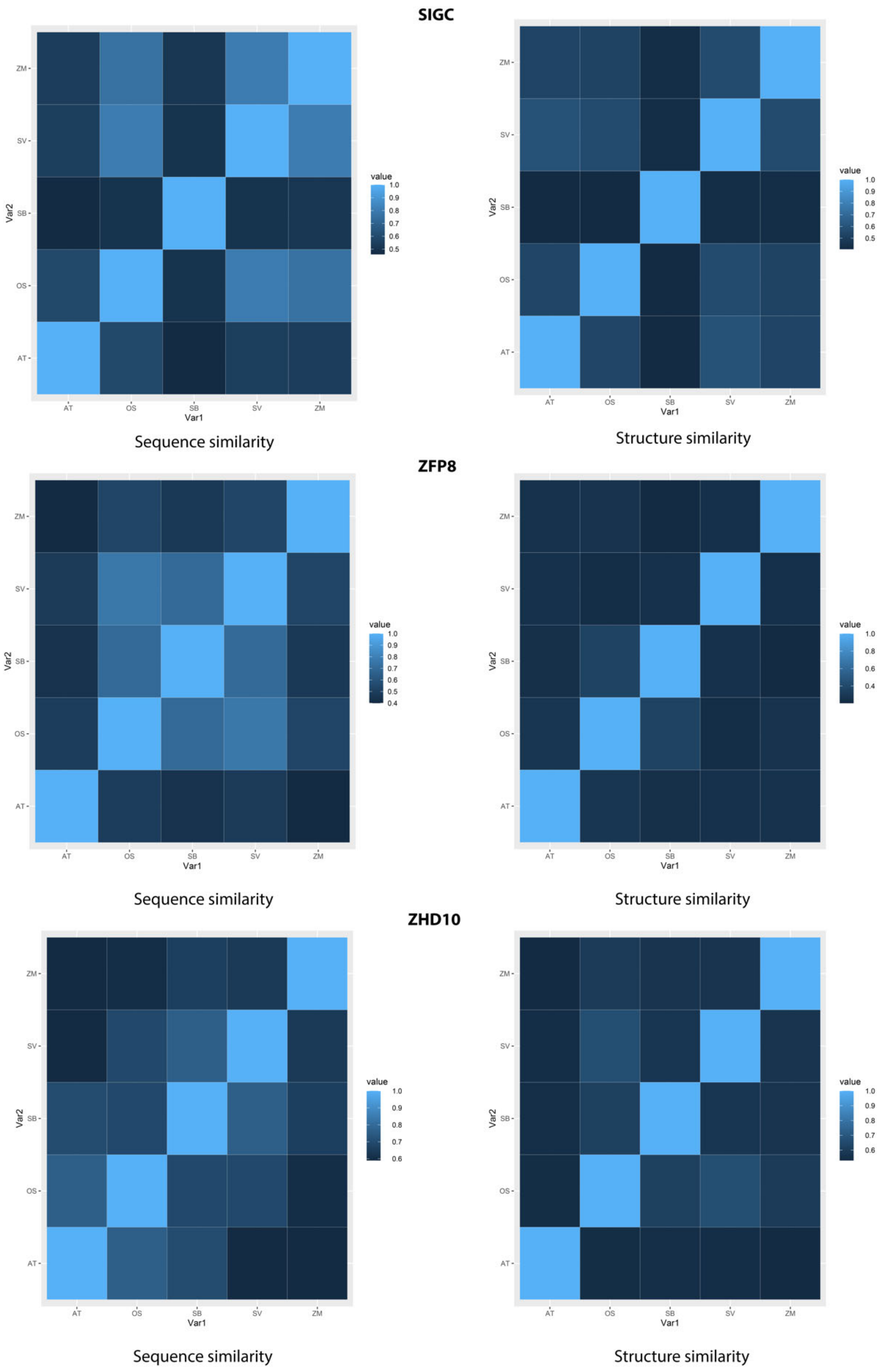

Figure S3. Sequence- and structure- similarities comparison of 3 transcription factor type FOG proteins identified by Aubry *et al.*

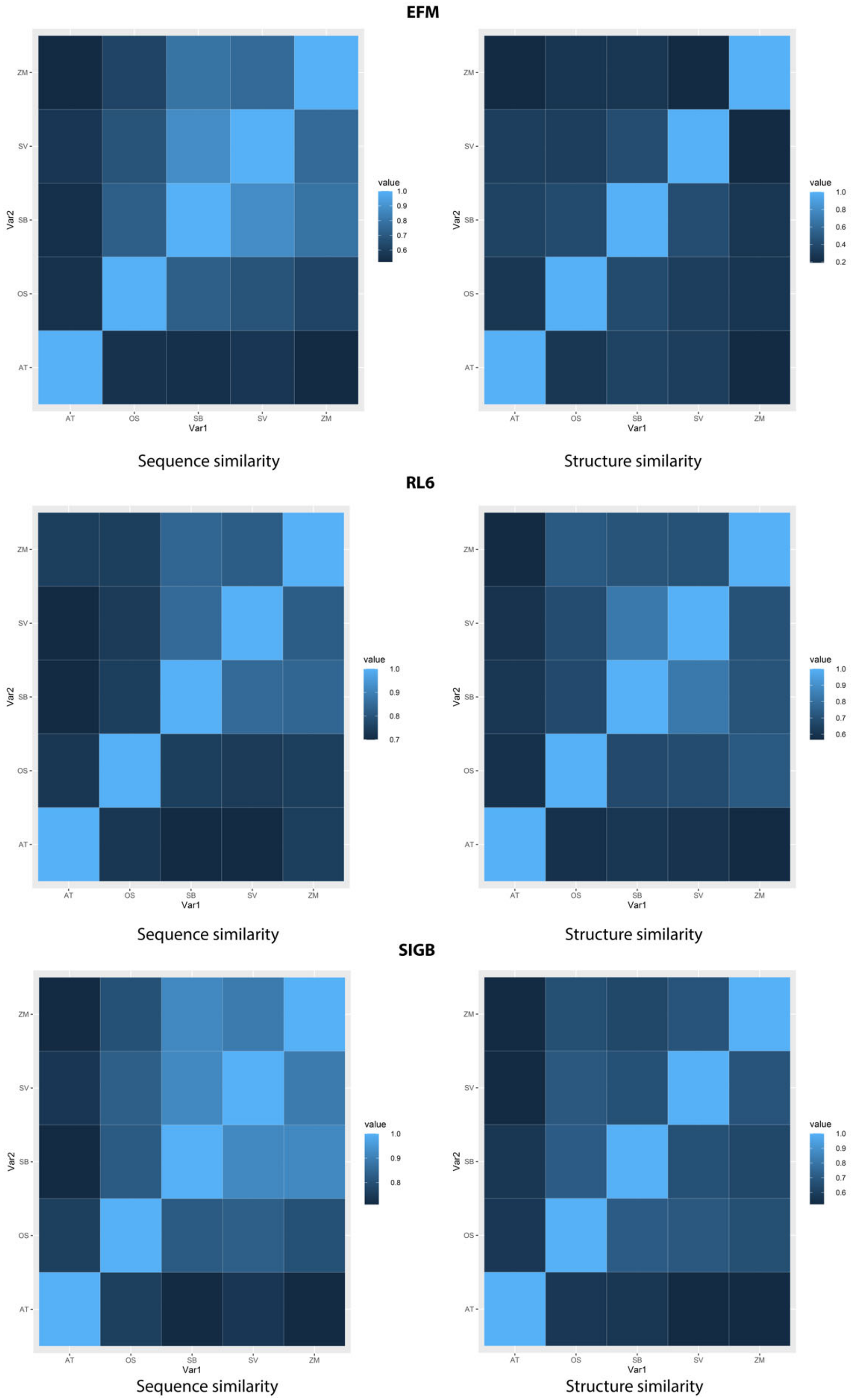

Figure S4. Sequence- and structure- similarities comparison of 3 transcription factor type non-FOG proteins identified by Aubry *et al*.
